# Older adults’ awareness of modifiable risk and protective factors for dementia and interest in eHealth interventions for brain health: a comparison between the Netherlands and Germany

**DOI:** 10.1186/s12889-023-17247-6

**Published:** 2023-11-23

**Authors:** Andrea E. Zülke, Melanie Luppa, Martin van Boxtel, Kay Deckers, Irene Heger, Sebastian Köhler, Steffi G. Riedel-Heller

**Affiliations:** 1 Institute of Social Medicine, Occupational Health and Public Health (ISAP), University of Leipzig, Leipzig, Germany; 2 Alzheimer Centrum Limburg, Department of Psychiatry and Neuropsychology, School for Mental Health and Neuroscience (MHeNs), Maastricht University, Maastricht, the Netherlands

**Keywords:** Dementia, Alzheimer Disease, Prevention, Risk factor, Lifestyle, Brain health, Survey, eHealth

## Abstract

**Background:**

Evidence on modifiable risk factors for dementia is accumulating rapidly, including e.g. smoking, hypertension, and diabetes. Comparing knowledge of risk factors for dementia and factors associated with knowledge and motivation to learn about dementia risk reduction in different countries may support the design of tailored public health campaigns. We investigated (1) differences in knowledge of risk and protective factors for dementia between the Netherlands and Germany, and interest in (2) information on brain health and (3) eHealth for brain health.

**Materials and methods:**

Population-based telephone (Germany) or web-based surveys (Netherlands) were conducted among adults aged 60–75 (n_total_=614; Germany: n = 270; Netherlands: n = 344), assessing sociodemographic factors, knowledge of risk and protective factors for dementia, interest in information on brain health and respective eHealth-tools. Correlates of knowledge, interest in information on brain health and eHealth for brain health were analyzed using multivariable regression, by country and in pooled analyses.

**Results:**

In the total sample (M_age_: 67.3 (SD: 4.3) years; %_female_: 48.6), knowledge of risk and protective factors (sum score assessing number of correctly identified factors) was higher among German participants (M (SD) = 7.6 (2.5) vs. 6.0 (4.3), *p* < .001). This was confirmed using linear regression analyses, controlling for sociodemographic covariates (b = 1.51; 95% CI: 1.00; 2.01). High education was linked to better knowledge of risk and protective factors (b = 1.61; 95% CI: 0.89; 2.34). Controlling for covariates, interest in information on brain health (OR: 0.05, 95% CI: 0.02; 0.09) and eHealth for brain health (OR: 0.40, 95% CI: 0.25; 0.65) was lower in German participants. Widowed participants were less interested in information on brain health, while widowed and single participants expressed less interest in eHealth for brain health in pooled analyses. Further associations between sociodemographic factors, interest in information on brain health and eHealth for brain health by country were detected.

**Discussion:**

Engaging older adults in the design of eHealth interventions and cooperation with trusted sources, e.g., general practitioners, might enhance appreciation of eHealth for brain health. Education on risk and protective factors for dementia is warranted in both countries. However, differences in recruitment and assessment need to be acknowledged.

## Background

Globally, an estimated 55 million people are currently living with dementia, a number expected to increase up to 139 million in 2050 [1]. According to Alzheimer Europe, the number of people living with dementia in the Netherlands will increase from more than 250,000 (1.5% of the total population) in 2018 to over 540,000 in 2050 (3.2% of the total population). In Germany, respective numbers are expected to rise from 1,585,166 persons (1.9% of the population) in 2018 to more than 2,7 million (3.4% of the total population) in 2050 [2]. These numbers indicate a very urgent need for strategies for risk reduction, e.g., by altering modifiable risk factors for dementia. Research on risk factors for dementia that are amendable to change has increased tremendously in the last years. The Lancet Commission on Dementia Prevention, Intervention and Care highlighted 12 modifiable risk factors in its 2020 report (low education, hearing loss, traumatic brain injury, hypertension, obesity, high alcohol consumption (*>* 21 units per week), diabetes mellitus, depression, physical inactivity, smoking, social isolation, exposure to air pollution; [3]). Further evidence suggests detrimental effects of, e.g., stress [4] or sleep disturbances [5] on risk for dementia.

To maximize risk reduction potential, it is crucial that the general population is aware of established risk factors for dementia. Improving dementia literacy is stressed as a priority by the World Health Organization in its global status report on the public health response to dementia [6]. However, it appears that knowledge of modifiable risk and protective factors for dementia is low in the general population. A systematic review of international studies (total n of participants: 36,519) found that about 50% of respondents believed dementia to be a part of the natural ageing process [7]. In a global survey conducted by Alzheimer’s Disease International among more than 70,000 people, this belief was held by even 70% of respondents [8]. Especially, knowledge on cardiovascular/metabolic risk factors for dementia was found to be low [9, 10], while a recent review reported slightly better knowledge of modifiable risk factors in more recent studies [11].

Assessing the general population’s knowledge of risk and protective factors is crucial to inform public health campaigns and identify needs for tailored education and interventions for dementia risk reduction. To date, studies comparing the state of knowledge on risk factors for dementia between different countries or settings with comparable instruments are scarce, limiting direct comparisons between countries. Comparing knowledge of specific risk and protective factors for dementia between different countries and illuminating factors associated with knowledge and interest in the topic may help inform tailored public health approaches towards dementia risk reduction. If public knowledge of risk factors for dementia, and interest in the topic of brain health differs between countries, different or adapted strategies might be needed to raise awareness and motivate older adults to engage in brain healthy behavior. In other words, respective evidence may be helpful to determine whether the same approach may be readily implemented in different settings, or which modifications might be necessary to adapt strategies for dementia risk reduction to different national contexts.

Germany and the Netherlands share many similarities in terms of population structure and healthcare. Median population age was slightly higher in Germany (45.8 years) than in the Netherlands (42.7 years) in 2022. On the other hand, the share of people aged 65 and older increased more strongly in the Netherlands than in Germany between 2012 and 2022 (3.8% vs. 1.4%, respectively; [12]). While overall healthcare expenditure is slightly higher in Germany (12.7% of gross domestic product (GDP); the Netherlands: 11.2% of GDP), expenditure on means of preventive healthcare is higher in the Netherlands (0.51% of GDP vs. 0.41% in Germany, respectively) [13].

Electronic health (eHealth) interventions provide a promising approach to disseminate means of dementia risk reduction to a larger public. Respective interventions provide several benefits, e.g., independent usage, low-threshold accessibility, a high degree of personalization, and possibly outreach to underserved populations, such as older adults living in rural areas with limited access to healthcare. Beneficial effects of eHealth interventions for older adults have been reported on several outcomes, including physical activity [14], healthy eating and blood pressure control in a systematic review [15]. Small to moderate effects of multidomain eHealth interventions on cognitive outcomes (global cognition, subjective cognitive function, dementia risk) were observed in a systematic review and meta-analysis [16]. In the Netherlands, a digital intervention to provide information on lifestyle and brain health and promote brain-healthy behavior change has recently been implemented and evaluated in a proof-of-concept-study [17]. Respective eHealth interventions for dementia risk reduction in Germany are currently lacking.

Against this background, we aim to (1) compare levels of knowledge of established risk and protective factors for dementia, (2) assess interest in information on dementia risk reduction and use of eHealth interventions for brain health in a sample of older adults from Germany and the Netherlands.

## Methods

### Participants

#### Germany

Participants in the German survey were interviewed via telephone between March and April 2022. The survey was part of a research project investigating dementia literacy in the older general population as well as interest in eHealth applications for brain health. Computer-assisted telephone interviews were conducted by trained interviewers of USUMA GmbH, a social research institute based in Berlin with expertise in conducting health-related research. Eligible participants had to be aged 60 or older and living in a private household in Germany, the targeted sample size being n = 500. A multi-stage random digit dialing procedure was applied, drawing upon the sample base of the Association of German Market and Social Research Agency (ADM). The resulting sample included registered and non-registered landline telephone numbers from the German resident population. Telephone numbers were selected proportionately to the German population structure, stratified regionally accounting for differences in district sizes. The Kish-Selection-Grid was applied if households with more than one person ≥ 60 years were selected. A researcher of Leipzig University trained all interviewers prior to study enrollment, and interviewers were randomly monitored for quality control. Further details on the sample can be found elsewhere [10].

#### The Netherlands

The survey conducted in the Netherlands was part of the *MijnBreincoach* („MyBraincoach“ in English)-project. This project consists of a public health campaign, developed by the Alzheimer Centrum Limburg of Maastricht University. The data used for the present study includes the pre-campaign levels of knowledge of risk and protective factors for dementia, assessed using an online-survey among community-dwelling adults aged 40 to 75 years in September 2017. Participants were recruited in two ways: First, participants from a previous national health survey who had agreed to be re-contacted for future research and were living in the Province of Limburg received an invitation to participate via e-mail. Second, participants were recruited via “living labs” conducted in the course of the project in the towns of Brunssum, Landgraaf and Roermond. Respective participants were randomly selected based on ZIP-code and age and were invited via mailed invitations. Recruitment procedures are further described in [18].

The initial sample included n = 1,090 persons (Germany: n = 500, the Netherlands: n = 590). In the German survey, participants aged 60 years and older were included, while the Dutch study surveyed adults aged 40 to 75 years. To ensure comparable samples with regard to age, participants younger than 60 years (n = 245) and older than 75 years (n = 231) were excluded for the current study. Therefore, the final analysis sample contained n = 614 persons (Germany: n = 270; the Netherlands: n = 344).

### Measures

#### Sociodemographic information

German participants provided information on sex, age, relationship status and education during the telephone interviews, assessed using a standardized questionnaire. Responses were documented electronically by USUMA GmbH. In the Dutch survey, participants received a letter or e-mail containing login-data to the web-based survey, where they provided information on age, sex, relationship status and educational attainment.

#### Knowledge of risk factors for dementia and attitudes towards dementia risk reduction

Participants’ knowledge of risk and protective factors for dementia was assessed using a standardized questionnaire, developed by Heger and colleagues [18]. Risk and protective factors were presented as closed-ended statements, e.g., “High blood pressure increases the risk for dementia”, and response options (“strongly agree”, “agree”, “neither agree nor disagree”, “disagree”, “strongly disagree”) were displayed using a 5-point Likert-scale. The questionnaire assessed the following established risk and protective factors for dementia: hypertension, hypercholesterolemia, obesity, chronic kidney disease, coronary heart disease, physical activity, depression, diabetes mellitus, smoking, cognitive activity, low to moderate alcohol consumption, healthy diet. Additionally, both surveys included two sham-items assessing factors not associated with dementia risk (poor personal hygiene; having children) to detect monotone answering tendencies. We calculated a sum score, with one point given for each correctly identified risk or protective factor and correctly refused sham-item, higher scores indicating better knowledge of risk and protective factors for dementia.

Further, we assessed self-rated knowledge about dementia (Dutch survey: “How much would you say you know about dementia?”, response options: “a great deal”, “quite a lot”, “some”, “not very much”, “nothing at all”, “I don’t know”; German survey: “How would you rate your knowledge on dementia – would you say you know…”; response options: “very much”, ”a lot”, “something”, “rather little”, “nothing”, “I don’t know”). To assess interest in dementia risk reduction, participants answered the question “Would you be interested in receiving information on how to improve your brain health?“, response options: „yes“, „maybe“, „no“. Participants indicated whether they knew/had known someone with dementia, choosing among the following options (multiple entries possible): a partner, parent or child; a grandparent; a friend or acquaintance; a colleague/somebody at work; someone else; nobody. The surveys further investigated interest in eHealth/mHealth interventions for brain health (“In the case that there was a mobile application (German survey: “that means a website or an app”), providing you without charge with information about your brain health and giving advice on how to improve your brain health, would you use this app?”). The respective response options were “yes”, “maybe”, “no”.

### Statistical analyses

Observations from Germany and the Netherlands were compared on sociodemographic characteristics and knowledge of established risk or protective factors for dementia using Chi²- and t-tests, as appropriate. We assessed factors associated with (1) interest in information on brain health, (2) interest in using eHealth for brain health using multivariable logistic regression, controlling for differences in age, sex, level of education, marital status, self-rated knowledge of dementia and country of study. To account for systematic differences between samples, observations from the two surveys were matched on age, sex, education, marital status and whether participants knew/had known a person with dementia (yes or no) using entropy balancing. This non-parametric weighting approach matches observations from one sample (e.g., Germany) to observations from a control group (here: the Netherlands) which is comparable in terms of pre-specified observable characteristics [19]. Sample characteristics are provided using unmatched observations, while comparisons in knowledge of risk and protective factors and multivariable regression analyses were conducted using entropy balancing to account for systematic differences in sociodemographic characteristics between samples. A significance level of *p* = .05 was applied (two-tailed tests). All analyses were conducted using Stata 16.0 (SE).

## Results

**Fig. 1:**
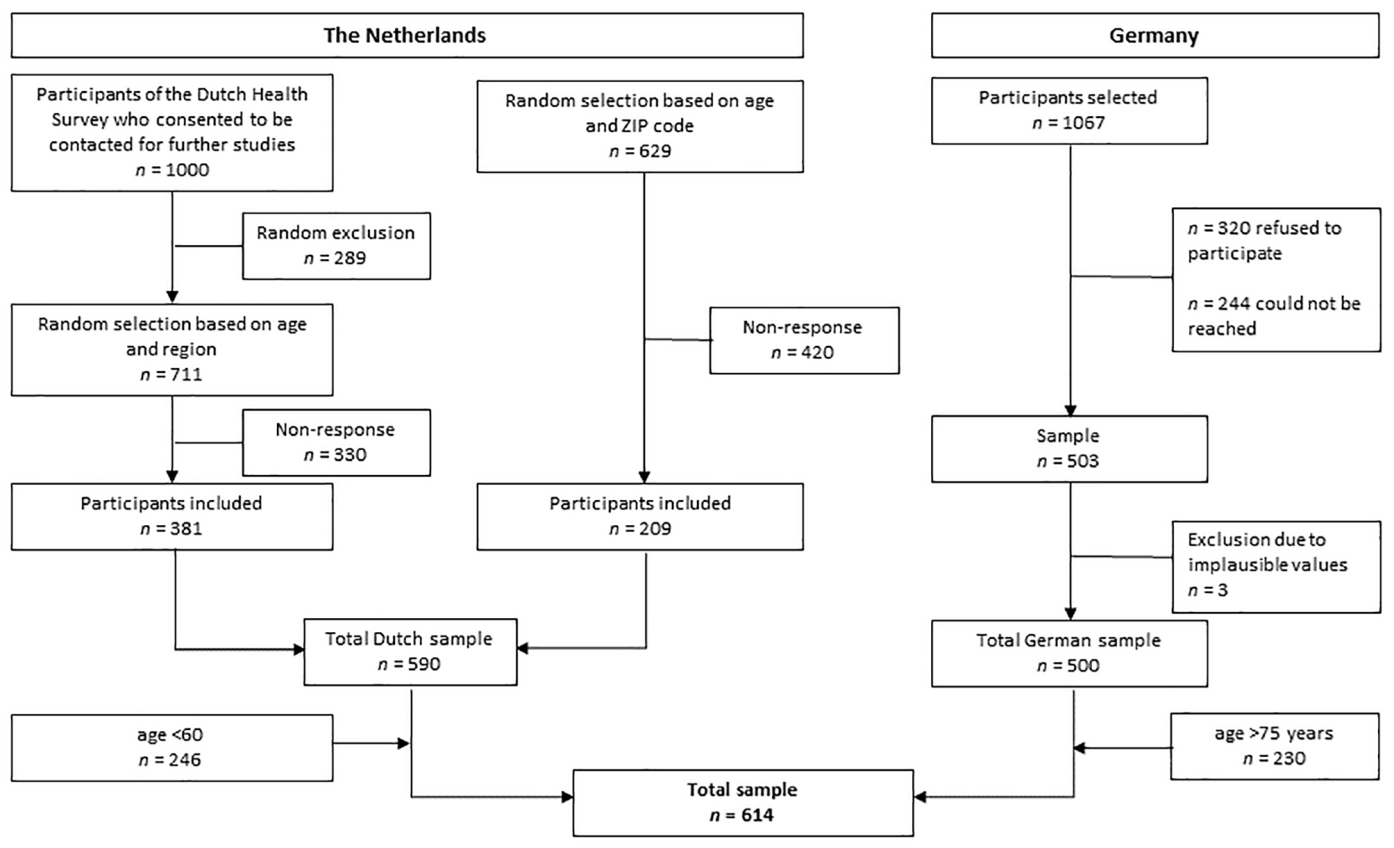
Recruitment of participants in the Dutch and German survey

The recruitment process for participants of both surveys is displayed in Fig. 1.

### Sample characteristics

Sociodemographic characteristics of participants in both surveys, as well as self-rated knowledge of dementia, are given in Table 1.

**Table 1:** Sociodemographic characteristics and self-rated knowledge on dementia of participants in Germany and the Netherlands

	Germany (n = 270)	The Netherlands (n = 344)	*p*
	% (n) / M (SD)		
Age, years	67.7 (4.3)	66.9 (4.4)	0.025
Female sex (ref.: male)	58.9 (159)	40.4 (137)	< 0.001
Education			
Low	13.4 (36)	20.2 (69)	0.068
Intermediate	39.9 (107)	39.3 (134)	
High	46.6 (125)	40.5 (138)	
Marital status			
Married/in a relationship	53.0 (143)	79.8 (272)	< 0.001
Single	18.2 (49)	3.8 (13)	
Divorced	12.6 (34)	7.0 (24)	
Widowed	16.3 (44)	9.4 (32)	
Self-rated knowledge of dementia			
Excellent / good	54.4 (147)	39.5 (132)	0.001
Intermediate	35.2 (95)	47.9 (160)	
Insufficient / none	10.4 (28)	12.6 (42)	
Knowing someone with dementia (yes)	73.3 (198)	86.1 (296)	< 0.001

### Knowledge of risk and protective factors for dementia

Rates of correctly identified risk and protective factors for dementia by country, using (1) unmatched data and (2) matched data using entropy balancing to account for sociodemographic differences between samples, are described in Table 2. As protective factors, cognitive and physical activity, a healthy diet and low to moderate alcohol consumption were identified correctly more often in the German than in the Dutch sample. Further, the risk factors depression, smoking and elevated cholesterol were endorsed more often among German participants. However, German participants more often (incorrectly) believed that poor personal hygiene increases risk for dementia. Endorsement of hypertension, obesity, heart disease, chronic kidney disease and having children (sham-item) did not vary by country. In Germany, 63.0% disagreed or disagreed strongly that “there is nothing I can do to reduce my dementia risk”, indicating belief in the possibility of dementia risk reduction, while the respective proportion was 39.9% in the Netherlands (*p* < .001; not tabulated).

**Table 2:** Correctly identified risk/protective factors for dementia, by country (percent)

	unadjusted			adjusted			Missings (Germany / Netherlands), %
	Germany (n = 270)	The Netherlands (n = 344)	**p**	Germany (n = 270)	The Netherlands (n = 344)	*p*	
	endorsement, %	endorsement, %		endorsement, %	endorsement, %		
Cognitive activity	88.8	80.2	**0.005**	88.7	81.7	**0.042**	1.1/4.4
Physical activity	86.8	65.9	**< 0.001**	86.7	63.1	**< 0.001**	1.5/3.8
Healthy diet	66.5	51.4	**< 0.001**	66.3	48.5	**< 0.001**	1.5/4.4
Depression	63.5	42.3	**< 0.001**	63.2	40.6	**< 0.001**	3.7/3.8
Low/moderate alcohol consumption	63.4	27.0	**< 0.001**	63.1	26.4	**< 0.001**	3.0/3.2
Smoking	49.2	35.7	**0.001**	48.8	32.8	**0.001**	7.4/3.2
Elevated cholesterol	34.9	25.8	**0.020**	34.8	20.2	**< 0.001**	11.9/4.4
Hypertension	34.0	30.6	0.390	33.9	27.7	0.198	10.7/3.2
Obesity	28.4	25.1	0.364	28.2	21.1	0.085	4.8/3.8
Heart disease	18.3	15.5	0.375	18.4	13.0	0.129	8.9/4.4
Poor personal hygiene (sham item)	16.2	6.3	**< 0.001**	**15.9**	**7.4**	**0.014**	3.7/3.8
Chronic kidney disease	12.0	9.4	0.330	12.1	11.1	0.766	16.7/4.4
Having children (sham item)	2.3	0.6	0.079	2.3	0.2	**< 0.001**	3.0/4.4
Number of correctly identified risk/protective factors, mean (SD)	7.6 (2.5)	6.2 (3.2)	**< 0.001**	7.6 (2.5)	6.0 (4.3)	**< 0.001**	

Response options “agree strongly” and “agree” indicate endorsement of risk/protective factors; significant differences highlighted in bold type

In a next step, overall-knowledge of risk and protective factors for dementia, assessed using a sum-score of correctly identified risk and protective factors, was assessed using multivariable linear regression (Table 3). Among the German subsample, women had poorer knowledge of risk and protective factors for dementia (b = -0.69; 95% CI: -1.32; -0.06), while those with a high level of education had better knowledge (b = 1.14, 95% CI: 0.27; 2.00). For Dutch participants, intermediate (b = 0.99; 95% CI: 0.01; 1.98) and high levels of education (b = 2.19; 95% CI: 1.21; 3.18) were linked to better knowledge of risk and protective factors. Participants from Germany identified more risk and protective factors for dementia correctly (b = 1.51, 95% CI: 1.00-2.01). Further, a high level of education was linked to better knowledge of risk and protective factors for dementia (b = 1.61, 95% CI: 0.89; 2.34). Knowledge of risk and protective factors for dementia was not linked to sex, age, marital status, self-rated knowledge about dementia or knowing a person with dementia in pooled analyses.

**Table 3:** Knowledge of modifiable risk and protective factors for dementia, linear regression

	Overall (n = 600)			Germany (n = 268)			The Netherlands (n = 332)		
	Coeff.	95% CI	*p*	Coeff.	95% CI	*p*	Coeff.	95% CI	*p*
Country (ref.: the Netherlands)	1.51	1.00; 2.01	**< 0.001**	-	-	-	-	-	-
Age	-0.04	-0.09; 0.02	0.167	-0.04	-0.11; 0.02	0.176	-0.06	-0.15; 0.02	0.147
Female sex (ref.: male)	-0.44	-0.94; 0.07	0.091	-0.69	-1.32; -0.06	**0.032**	-0.27	-1.06; 0.51	0.497
Education (ref.: low)									
Intermediate	0.58	-0.11; 1.28	0.098	0.08	-0.79; 0.95	0.857	0.99	0.01; 1.98	**0.048**
high	1.61	0.89; 2.34	**< 0.001**	1.14	0.27; 2.00	**0.010**	2.19	1.21; 3.18	**< 0.001**
Marital status (ref.: married/in a relationship)									
Single	-0.79	-1.63; 0.05	0.064	-0.29	-1.16; 0.58	0.512	-1.27	-2.65; 0.012	0.073
Divorced	-0.38	-1.23; 0.47	0.381	0.33	-0.62; 1.28	0.498	-1.10	-2.44; 0.25	0.109
Widowed	0.04	-0.72; 0.80	0.916	-0.25	-1.14; 0.64	0.581	0.39	-0.80; 1.57	0.520
Self-rated knowledge about dementia (ref.: low)									
Intermediate	0.15	-0.79; 1.10	0.750	-0.39	-1.53; 0.76	0.506	0.70	-0.69; 2.09	0.325
High	0.52	-0.40; 1.44	0.268	0.00	-1.09; 1.08	0.994	1.22	-0.18; 2.62	0.087
Knowing someone with dementia (yes)	0.31	-0.31; 0.92	0.332	-0.19	-0.88; 0.50	0.592	0.78	-0.16; 1.71	0.103

Outcome: sum-score of correctly identified modifiable risk and protective factors for dementia (for sham-items “having children” and “poor personal hygiene”: one point given for correct refusal); range: 0–14. Higher scores indicate better knowledge of risk and protective factors; significant associations highlighted in bold type; CI: confidence interval; Coeff: coefficient

### Interest in information on brain health

Asked whether they were interested in receiving further information on how to promote brain health, 20.4% of German participants stated “yes”, with further 16.3% stating “maybe”. In the Dutch subsample, 75.2% were interested in respective information, and further 16.2% answered “maybe” (*p* < .001). Further, differences regarding preferred source of information were detected: while 58.7% of Dutch participants would search the Internet for information on brain health, only 24.1% of German participants endorsed this option (not tabulated). Controlling for covariates, German participants who were widowed were less likely to be interested in information on brain health (OR: 0.39; 95% CI: 0.19; 0.96; Table 4). In the Dutch subsample, no associations between sociodemographic characteristics, knowledge of risk and protective factors or self-assessed knowledge of dementia and interest in information on brain health were detected. Pooling observations from both countries (overall-model), participants from Germany were less interested in information on brain health (OR: 0.05; 95% CI: 0.02; 0.09). Further, participants who were widowed were less likely to be interested in respective information in the overall-model (OR: 0.45; 95% CI: 0.22; 0.91).

**Table 4:** Interest in information on brain health, logistic regression

	Overall (n = 577)			Germany (n = 268)			The Netherlands (n = 309)		
	OR	95% CI	*p*	OR	95% CI	*p*	OR	95% CI	*p*
Country (ref.: the Netherlands)	0.05	0.02; 0.09	**< 0.001**	-	-	-	-	-	-
Age	1.03	0.98; 1.09	0.273	1.05	0.99; 1.12	0.127	1.01	0.91; 1.12	0.875
Female sex (ref.: male)	0.79	0.48; 1.32	0.376	0.70	0.39; 1.23	0.215	1.28	0.42; 3.89	0.666
Education (ref.: low)									
Intermediate	0.51	0.24; 1.12	0.095	0.47	0.21; 1.07	0.071	0.99	0.25; 4.01	0.994
high	0.86	0.42; 1.78	0.691	0.63	0.28; 1.39	0.247	3.28	0.55; 19.56	0.192
Marital status (ref.: married/in a relationship)									
Single	0.62	0.26; 1.46	0.272	0.90	0.45; 1.84	0.781	0.22	0.04; 1.10	0.065
Divorced	0.66	0.36; 1.23	0.195	0.46	0.19; 1.11	0.085	3.45	0.37;32.18	0.277
Widowed	0.45	0.22; 0.92	**0.029**	0.43	0.19; 0.96	**0.039**	0.38	0.10; 1.53	0.174
Self-rated knowledge about dementia (ref.: low)									
Intermediate	2.24	0.89; 5.66	0.087	1.50	0.57; 3.94	0.411	4.06	0.61; 26.98	0.147
High	1.65	0.60; 4.51	0.327	1.29	0.49; 3.38	0.610	1.61	0.27; 9.43	0.598
Knowing someone with dementia (yes)	1.01	0.57; 1.79	0.979	1.54	0.79; 3.03	0.205	0.33	0.07; 1.52	0.155
Knowledge of risk and protective factors (sum score)	1.08	0.98; 1.20	0.127	1.09	0.97; 1.23	0.135	1.06	0.86; 1.32	0.568

Outcome: interest in information on brain health (response options “yes” and “maybe” vs. “no”). Significant associations highlighted in bold type; CI: confidence interval; OR: Odds Ratio

### Appreciation of eHealth for brain health

Among German participants, 36.3% stated interest in using an eHealth tool for brain health, with further 18.5% willing to consider it. The respective proportions were 51.1% and 27.4% in the Dutch subsample (*p* < .001; not tabulated). In logistic regression analyses, German participants who were either divorced (OR: 0.38; 95% CI: 0.17; 0.86) or widowed (OR: 0.27; 95% CI: 0.12; 0.60) were less likely to consider use of eHealth for brain health (Table 5). Those who knew/had known a person with dementia (OR: 2.24; 95% CI: 1.19; 4.22) or had better knowledge of risk and protective factors for dementia (OR: 1.18; 95% CI: 1.05; 1.32) were more likely to be interested in respective tools. Among the Dutch subsample, older age (OR: 0.89; 95% CI: 0.81; 0.99) and being single (OR: 0.13; 95% CI: 0.04; 0.49) was linked to lower interest in eHealth for brain health. In the overall-model, including “country” as covariate, German participants were less likely to be interested in eHealth for brain health (OR: 0.35; 95% CI: 0.21; 0.58), as were participants who were single (OR: 0.35; 95% CI: 0.16; 0.75) or widowed (OR: 0.53; 95% CI: 0.29; 0.96).

**Table 5:** Interest in eHealth for brain health, logistic regression

	Overall (n = 577)			Germany (n = 268)			The Netherlands (n = 309)		
	OR	95% CI	*p*	OR	95% CI	*p*	OR	95% CI	*p*
Country (ref.: the Netherlands)	0.35	0.21; 0.58	**< 0.001**	-	-	-	-	-	-
Age	0.97	0.92; 1.03	0.291	1.03	0.97; 1.10	0.356	0.89	0.81; 0.99	**0.026**
Female sex (ref.: male)	0.95	0.60; 1.51	0.828	1.31	0.74; 2.32	0.356	0.88	0.40; 1.93	0.752
Education (ref.: low)									
Intermediate	0.98	0.52; 1.82	0.938	1.02	0.44; 2.33	0.966	1.32	0.48; 3.69	0.590
high	1.13	0.60; 2.11	0.711	1.42	0.62; 3.28	0.407	1.42	0.48; 4.18	0.529
Marital status (ref.: married/in a relationship)									
Single	0.35	0.16; 0.75	**0.007**	0.65	0.32; 1.30	0.223	0.13	0.04; 0.49	**0.002**
Divorced	0.52	0.27; 1.02	0.056	0.38	0.17; 0.86	**0.019**	0.45	0.14; 1.45	0.182
Widowed	0.53	0.29; 0.96	**0.037**	0.27	0.12; 0.60	**0.001**	1.23	0.38; 3.96	0.727
Self-rated knowledge about dementia (ref.: low)									
Intermediate	0.90	0.43; 1.91	0.791	1.28	0.50; 3.30	0.610	0.43	0.09; 1.97	0.276
High	1.06	0.49; 2.28	0.879	0.96	0.37; 2.54	0.942	0.83	0.17; 4.02	0.815
Knowing someone with dementia (yes)	1.38	0.80; 2.36	0.244	2.24	1.19; 4.22	**0.013**	1.07	0.41; 2.78	0.892
Knowledge of risk and protective factors (sum score)	1.09	1.00; 1.19	0.063	1.18	1.05; 1.32	**0.005**	1.01	0.85; 1.20	0.908

Outcome: interest in information on brain health (response options “yes” and “maybe” vs. “no”). Significant associations highlighted in bold type; CI: confidence interval; OR: Odds Ratio.

## Discussion

Our study assessed the current state of knowledge on established modifiable risk and protective factors for dementia in two European countries, i.e., the Netherlands and Germany. We aimed to describe (1) differences between countries in knowledge of modifiable risk and protective factors, (2) differences in older adults’ interest in further information on brain health and openness towards respective eHealth-interventions between countries, and (3) other participant characteristics associated with better knowledge or interest.

### Knowledge of risk and protective factors for dementia

In both Germany and the Netherlands, endorsement of risk and protective factors was highest for lifestyle-related behaviors, i.e., cognitive and physical activity and a healthy diet. However, rates of endorsement were higher for each of these factors in the German subsample. Our results indicate the need for more information on the links between cardiovascular and metabolic conditions and dementia in both countries. Controlling for differences in age, sex, education, marital status, self-rated knowledge about dementia and knowing someone with dementia, overall knowledge of risk and protective factors was higher in the German subsample, as indicated by linear regression analyses. A high level of education was associated with better knowledge of risk and protective factors for dementia. These findings are in line with previous reviews and meta-analyses on dementia literacy, indicating that knowledge of risk and protective factors is fair to moderate overall [7].

Still, it cannot be ruled out that respective advantages of German participants may, in part, be due to methodological aspects. Social desirability, which affects telephone-based surveys more strongly than web-based assessments [20], may have led to higher endorsement of presented risk and protective factors. This could also explain higher endorsement of sham-items, i.e., having children and poor personal hygiene, in the German subsample. What is more, increased attention was given to the topic of dementia risk reduction during the time between the two surveys, e.g., by publication of the 2020-report of the Lancet Commission on dementia prevention, intervention and care [3] or the WHO’s guidelines on risk reduction of cognitive decline and dementia in 2019 [21] and respective media coverage. This may have provided an advantage for German participants regarding information on risk factors for dementia. The results are unlikely explained by higher levels of health literacy in general, as a comprehensive overview of European countries reported higher levels of health literacy in the Netherlands than in Germany [22], and further findings even found decreased health literacy in Germany throughout the last decade [23].

### Interest in information on brain health

Interest in receiving information on brain health was lower in the German subsample, controlling for covariates. The German Federal Ministry for Families, Seniors, Women and Youth mentions the importance of dementia risk reduction, e.g., reducing smoking, physical inactivity and preventing cardiovascular diseases by preventive home-visits in its national strategy on dementia [24]. However, to date this strategy focusses rather on raising awareness for people living with dementia than on large-scale public health approaches towards dementia risk reduction. Respective public health campaigns to raise awareness for dementia risk reduction have been implemented in several European countries, including Ireland and the Netherlands. Evaluations revealed increased awareness for the protective factors physical activity and healthy nutrition in the Netherlands [25], and for dementia risk reduction by lifestyle change overall in Ireland [26]. Information on dementia risk reduction for the older population should take into account different needs and wishes of older adults, and also reluctance to engage with brain health and dementia. This could include positive framing of the topic and avoidance of scare-mongering language, e.g., by using terms like “brain health” or “healthy ageing” rather than “dementia risk”. Including senior citizens’ organizations, as well as local and national expert panels on dementia (e.g., the German Alzheimer Association) or older adults from the community in the design and/or implementation of respective campaigns and educational materials could possibly increase acceptance among the older population in Germany and illuminate what motivates older people to get informed about dementia risk reduction. To disseminate the message that “what is good for the heart is also good for the brain”, addressing dementia risk reduction in disease management programs (DMPs) for conditions that increase dementia risk, e.g., coronary heart disease and hypertension, might be a suitable way to reach older adults that do not express explicit interest in brain health. More research is needed to better understand what motivates or discourages older adults to engage with brain health and dementia risk reduction, including barriers to healthcare and measures of prevention and health promotion.

Another possible explanation for lower interest in German participants relates to the COVID-19 pandemic. During the pandemic, people were confronted with ubiquitous health information, often disseminated via the internet, with information changing rapidly. Certain studies have found evidence for information fatigue (i.e., exhaustion from prolonged exposure to health-related information beyond what is desired [27]) regarding health information in the course of the pandemic [28–30]. Therefore, interest in health information and digital means of health promotion and disease prevention might have been generally lower in 2022, when German participants were interviewed.

Widowed participants were less likely to be interested in respective information in both pooled analyses and the German subsample. Possibly, participants’ relationship status might have influenced perceived social support and motivation for healthy ageing and behavior change, with those being widowed experiencing less support or meaningful reasons to engage in further education on brain health. This finding is in line with a systematic review, reporting that older adults’ willingness to engage in health promoting activities is strongly influenced by support from families [31].

### Appreciation of eHealth interventions for brain health

Lastly, German participants were less likely to be interested in eHealth interventions for brain health than Dutch participants. Although statistics on internet usage in European countries support differences among older adults between the two countries (internet usage in the last three months (2020) among individuals aged 55–64, Netherlands: 93.2%, Germany: 91.7%; 65–74 years, Germany: 76.3%, Netherlands: 90.3%); [32], the observed difference in our data is striking. Our findings are, however, in line with a study by Merkel and Hess, based on Eurobarometer-survey data from 2017 on digital technologies across European countries [33]. Use of eHealth among people ≥ 65 amounted to 28.7% in Dutch survey participants, but only to 4.9% in older German adults. In a population-based survey on health app-use in older German adults (≥ 60 years of age), general use of health apps was low (16.5%), with the most important barriers towards usage being lack of trust and concerns about data protection [34]. Our findings for Germany are further underscored by a recent study, stating that GPs constitute the preferred sources of health information for 87.5% of Germans, while only 35.9% preferred information from the internet [23]. On another note, higher preferences for using the internet for information on brain health might partly be explained by differences in recruitment strategies: While participants in Germany were contacted via telephone, Dutch participants received invitations to the respective survey via e-mail, possibly indicating greater internet affinity in this subsample. This explanation is further supported by the finding that no age differences (< 65 years vs. ≥65 years) were found regarding appreciation of eHealth in the Dutch survey [18]. However, as the survey did not directly assess internet literacy or regular internet usage, this line of thought should be interpreted with caution.

Several strategies might increase acceptance of eHealth for brain health among older people. As previous studies pointed out lack of trust in the provider as barriers towards eHealth use in older adults [34–36], cooperation with older citizens’ organizations and expert consortia might help establish trust in respective approaches. Recommendation of eHealth tools by GPs might further increase confidence in and acceptance of eHealth tools [36]. Beyond that, including older adults’ feedback during app development has been highlighted an important feature to reduce barriers towards and increase acceptance of eHealth interventions [36]. For example, the Dutch MijnBreincoach-app, targeted at personalized dementia risk reduction, included individuals from the target population in development of the app and conducted a pilot test of the final product prior to dissemination [37]. Offering training and support in using eHealth tools has been pointed out as a facilitator of uptake and use of eHealth in older adults [36], and deemed necessary to avoid widening health inequalities, so that all older adults may benefit from eHealth interventions, regardless of socioeconomic status and level of internet literacy [38]. This could be facilitated by providing in-app support for technical difficulties, offering general training of older adults for using the internet for health-related questions, or by inclusion of relatives or the attending GP in initial training on eHealth devices.

Research on eHealth interventions for older adults suggests that, despite greater reluctance towards internet-based approaches than younger persons, older adults can benefit significantly from eHealth interventions for several health outcomes. Studies on eHealth for depressive disorders across different age groups repeatedly reported similar intervention benefit in older, compared to younger participants [39–41]. Interestingly, adherence to the intervention (e.g., frequency and duration of use, number of completed modules) was even better in older than in younger participants [41]. Recently, the Ambulatory Research in Cognition (ARC)-study conducted a smartphone-based study among older adults (60–93 years). Although higher age was linked to less smartphone use and higher reported difficulties regarding use of smartphones for several tasks, the enrollment rate was 86.7% and participants were highly adherent (smartphone-based cognitive assessments, conducted four times/day for seven consecutive days; median adherence: 85.7%). Adherence was independent of frequency of smartphone use or perceived difficulties regarding smartphones. Where necessary, the study personnel provided support regarding app-download, installation, or general smartphone use [42]. These findings suggest that eHealth tools can constitute an appealing offer for older adults, given adequate training and support and thoughtful implementation.

**Fig. 2:**
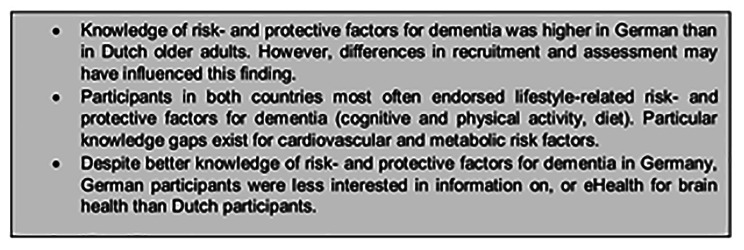
Summary of findings

Those who were not living in a relationship, i.e., who were single, divorced, or widowed expressed less interest in eHealth for brain health in our study. Possibly, social support brought about by an existing partnership increases motivation to engage in brain-healthy behavior. Additional effort might be necessary to raise interest in eHealth approaches to brain health in these groups, e.g., by emphasizing the benefits of health-promoting behavior for oneself and app-content providing appealing suggestions for activities and behaviors to be enjoyed alone. The main findings of the current study are summarized in Fig. 2.

### Strengths and limitations

By applying the same questionnaire in both surveys, we were able to draw direct comparisons regarding knowledge on risk factors for dementia, as well as interest in further information on brain health and the use of eHealth for brain health between two countries, drawing on a large population-based sample. Using closed-ended questions on established risk and protective factors facilitates the identification of specific gaps of knowledge and needs for further education on dementia risk reduction. The questionnaire assessing knowledge of risk and protective factors for dementia has further been used in other studies and different populations, e.g. in Norway [43], facilitating further cross-country comparisons.

However, the present study has several limitations. The method of assessment differed between the two surveys, with web-based questionnaires applied in the Netherlands and computer-assisted telephone interviews used in Germany. Social desirability and interviewer bias are known risks in telephone-based surveys [20], which may have skewed answering tendencies in the German subsample, possibly contributing to the higher number of correctly identified risk and protective factors in German participants. Further, the time-gap between the two surveys needs to be considered, with the Dutch and German surveys conducted in 2017 and 2022, respectively. During this time, the topic of dementia risk reduction has gained increased public attention, likely also attributable to increased numbers of scientific publications and respective communication of modifiable risk factors. This may have contributed to better knowledge of risk and protective factors observed in German participants. Regarding interest in eHealth for brain health, it cannot be ruled out that the Dutch sample was slightly more technology- and internet-literate than participants in the German survey, which may at least partially explain higher levels of interest in eHealth interventions observed among Dutch participants. The Dutch sample was recruited from participants of a prior health survey, possibly introducing selection bias by including participants with a general interest in health-related topics, which may have contributed to higher interest in information on brain health in the Dutch subsample. People with insufficient command of the Dutch/German language were excluded from participation, possibly introducing selection bias and making the results less generalizable to the general public in the two countries. Using closed-ended questions on risk and protective factors for dementia instead of asking open questions might have influenced participants’ answering tendencies. In previous studies applying open-ended questions, participants most often named cognitive activity or brain/memory training, physical activity and healthy nutrition as protective factors, whereas only few participants proactively named cardiovascular/metabolic risk factors [44–46]. However, two sham-items were included to control for monotone answering tendencies in order to enhance robustness of our findings. Lastly, our study compared observations from two high-income countries rather similar in terms of social structure, age distribution and internet access. Our results highlight that, despite high similarities between countries, prerequisites for public health campaigns to inform older adults on the links between lifestyle and brain health and for implementation of eHealth for brain health can differ remarkably. To the best of our knowledge, there are currently no other studies comparing interest in brain health and eHealth for brain health in different countries applying the same instruments, limiting comparability of our findings. Investigating dementia literacy and interest in information on brain health or eHealth for brain health between countries with more pronounced differences, e.g., between high- and low-to-middle income countries may further advance scientific efforts and shed light on possibilities to disseminate eHealth interventions for brain health in different local and cultural settings. To date, respective investigations, such as Alzheimer’s International’s global “Attitudes to dementia”-survey, are currently scarce [8].

## Conclusion

**Fig. 3:**
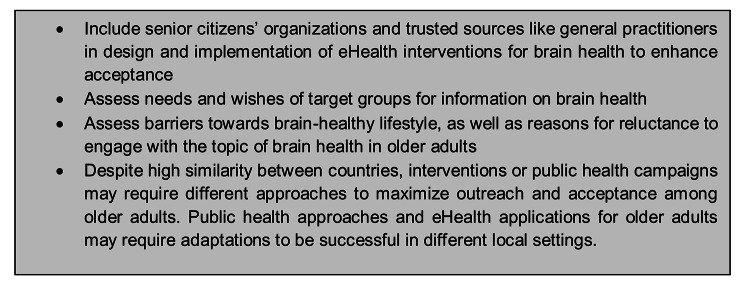
Recommendations for future studies

Our study identified significant differences in knowledge of risk and protective factors for dementia between older adults in the Netherlands and Germany. While knowledge of most risk and protective factors was slightly higher in German participants, a need for education especially on the role of cardiovascular and metabolic risk factors for dementia was evident in both countries. Both interest in receiving information on brain health and use of eHealth tools for brain health were lower in the German subsample, however, differences in recruitment and assessment may have contributed to these findings. These results highlight the need for more research on facilitators and barriers towards eHealth use in older German adults. Respective studies could inform the design of user-centered, effective eHealth interventions that take into account the needs and wishes of the target group. Inclusion of, e.g., general practitioners and senior citizens’ organizations in design and dissemination of eHealth tools for brain health might raise acceptance of respective approaches. Figure 3 summarizes recommendations for future studies.

## Data Availability

The dataset analyzed for this manuscript is available from the corresponding author upon reasonable request.

## List of Abbreviations

ADM: Association of German Market and Social Research Agency (Arbeitskreis deutscher Markt- und Sozialforschungsinstitute e.V.)
CI: Confidence interval
Coeff: Coefficient
GP: General practitioner
LIBRA: Lifestyle for BRAin Health
OR: Odds Ratio
SD: Standard deviation
WHO: World Health Organization
